# Under-reporting of inpatient services utilisation in household surveys – a population-based study in Hong Kong

**DOI:** 10.1186/1472-6963-5-31

**Published:** 2005-04-28

**Authors:** Eva LH Tsui, Gabriel M Leung, Pauline PS Woo, Sarah Choi, Su-Vui Lo

**Affiliations:** 1 Hospital Authority, 5/F, HA Building, 147B Argyle Street, Kowloon, Hong Kong, China; 2 Department of Community Medicine and School of Public Health, University of Hong Kong, 5/F, Academic & Administration Block, Faculty of Medicine Building, 21 Sassoon Road, Hong Kong, China; 3 Health Care Financing Study Group, Health and Welfare Bureau, Government of the Hong Kong Special Administrative Region, Murray Building, 3 Garden Road, Central, Hong Kong, China

## Abstract

**Background:**

Recognising that household interviews may produce biased estimates of health services utilisation, we examined for under- and over-reporting of hospitalisation episodes in three recent, consecutive population-based household surveys in Hong Kong.

**Methods:**

Territory-wide inpatient service utilisation volumes as estimated from the 1999, 2001 and 2002 Thematic Household Surveys (THS) were benchmarked against corresponding statistics derived from routine administrative databases. Between-year differences on net under-reporting were quantified by Cohen's d effect size. To assess the potential for systematic biases in under-reporting, age- and sex-specific net under-reporting rates within each survey year were computed and the F-test was performed to evaluate differences between demographic subgroups. We modelled the effects of age and sex on the likelihood of ever hospitalisation through logistic regression to compare the odds ratios respectively derived from survey and administrative data.

**Results:**

The extent of net under-reporting was moderately large in all three years amounting to about one-third of all inpatient episodes. However, there did not appear to be significant systematic biases in the degree of under-reporting by age or sex on stratified analyses and logistic regression modelling.

**Conclusion:**

Under-reporting was substantial in Hong Kong's THS. Recall bias was likely most responsible for such reporting inaccuracies. A proper full-design record-check study should be carried out to confirm the present findings.

## Background

Population and health services research commonly relies on in-person household interviews as the main source of health and health care data, in terms of disease, disability and utilisation of services. These types of information are important for evidence-based health policy formulation, planning and evaluation. While medical chart review, insurance claims records and government macro statistics are potential alternative sources of such information, they cannot entirely replace the household interview given the often prohibitive expense of data abstraction exercises, lack of population coverage of single data sources especially in a mixed medical economy where there is a multiplicity of financial intermediaries and care providers, and the inability to study individual-level associations with ecologic data respectively.

However, self-reported data from household survey reports are subject to various types of error. Generally, random reporting error tends to increase the variance and thus uncertainty associated with the data, whereas systematic reporting error can bias survey estimates. Therefore, it is important to study the accuracy and validity of data obtained from household surveys.

There are two broad categories of reporting error in surveys on health services utilisation: under-reporting and over-reporting. Under-reporting refers to respondents forgetting or otherwise omitting (often due to the sensitive nature of the questions that may reflect socially undesirable or embarrassing behaviour) relevant episodes. Over-reporting occurs when interviewees attribute episodes outside the reporting period or survey definition to their response. They may mis-report an episode outside the reference period as if it had happened within that period in either the forward or backward direction – i.e. the "telescoping" phenomenon which could also lead to over-reporting.

In Hong Kong where a mixed medical economy of public and private providers operate in parallel, with the former delivering over 94% of total bed-days and the latter responsible for more than 70% of all ambulatory episodes, household surveys have been the only viable and sustainable option to follow utilisation trends over time, the findings from which form the basis for health policy decisions. We therefore examined the accuracy and validity of recall in such recent surveys by comparing survey responses for inpatient utilisation to aggregate statistics from the two main public sector service organisations, namely the Hospital Authority (HA) for public inpatient data and corresponding figures on private hospital admissions collated by the Department of Health (DH).

The specific aims of the study were to (i) benchmark survey results against territory-wide macro estimates, based on administrative records, of the number of hospitalisation episodes in both the public and private sectors; (ii) analyse the variability in agreement between survey and administrative estimates with respect to age and sex; and (iii) consider the effect of reporting errors on the application of survey estimates for planning and research purposes.

## Methods

### Data sources

The Thematic Household Surveys (THS) are a regular series of cross-sectional in-person surveys of the land-based population of Hong Kong, conducted by the Census and Statistics Department (C&SD) of the Hong Kong Special Administrative Region Government, People's Republic of China. Different topics are covered in each round of THS. Topics related to health services utilisation such as hospitalisation and/or doctor consultation episodes had been surveyed 13 times since 1982. The three most recent such surveys were conducted in 1999, 2001 and 2002 consecutively [1-3]. The 2002 THS covered the entire land-based population of Hong Kong including both institutional and non-institutional residents whereas the 1999 and 2001 rounds only included the non-institutional population. (Table 1)

**Table 1:** Sample size and age-sex demographics of the last three rounds of THS

	**Sept – Nov 1999**	**Jan – May 2001**	**May – July 2002**
**No. of households successfully enumerated**	10,057	10,046	10,015 (and 2,111 institutional residents)
**Response rate**	77%	76%	78% (97%)

	**No. of individuals**	**No. of individuals**	**No. of individuals**
*Age (years)*			
Less than 5	1,506	1,298	1,108
5 – 14	4,682	4,385	3,849
15 – 24	4,875	4,808	3,974
25 – 34	5,471	5,418	4,360
35 – 44	6,684	6,472	5,774
45 – 54	4,420	4,856	4,551
55 – 64	2,514	2,493	2,524
65 or above	3,611	3,879	5,532
			
***Sex***			
Male	16,601	16,484	15,321
Female	17,162	17,125	16,351

**Total**	**33,763**	**33,609**	**31,672**

Respondents were asked to recall the total number of hospital admissions and associated characteristics (e.g. provider type, reason for attendance, payment details and so on) of each episode. The recall periods were 12 months in the 2002 THS, but six months for 1999 and 2001. In addition to details of inpatient care episodes, the surveys also collected information on demographic and socio-economic characteristics, health status and medical need (e.g. presence of chronic conditions, regular medications taken), medical benefits and insurance coverage, among others. Proxy reporting by primary caretakers was allowed for respondents aged 12 and below and those who were mentally unfit to respond to the survey, except for the self-reported health status questions.

Survey responses were compared, on an ecologic or macro level, with the "gold-standard" administrative databases maintained by DH and HA. The Hospital Authority, the dominant inpatient service provider (with >94% market share in terms of total bed-days), has detailed data on all inpatient episodes in the public sector and DH collects aggregate utilisation statistics routinely from all 12 private hospitals. Together, there is total coverage of hospitalization episodes in terms of volume of inpatient utilisation in the territory.

All inpatient administration procedures in all 44 public hospitals under the management of HA are electronically processed through an Integrated Patient Administration System (IPAS). The first version of this standardized corporate-wide system has been fully implemented since early 1994. It consists of (1) the Hong Kong Patient Master Index which is a corporate database holding personal particular details of patients; (2) admission/discharge/transfer modules which provide timely and comprehensive information on patient movement; and (3) a medical record indexing module which provides an indexing tool to facilitate the tracing and administration of patient medical records. All patients are identified by their Hong Kong resident identity card number. For non-residents, a pseudo identity card number is generated by the system and intended for repeated use in subsequent admissions. A unique episode number is generated for each inpatient episode within all HA hospitals, and coupled with the patients' identification card number form the common identifiers for all inpatient episodes within the HA system. Moreover, the system is linked to the patient billing system. Hong Kong has a very straightforward inpatient financing mechanism whereby the cost is 98% subsidised through general taxation as a one-line vote to the HA annual operating budget and the all-inclusive *per diem *point-of-care user fee at a public hospital is HK$100 or $68 prior to 2003 (HK$7.8 = US$1 pegged exchange rate) which is payable by all except for civil servants, HA staff and the socially and medically indigent (all certified by special entitlement cards captured in IPAS) through out-of-pocket payments without financial intermediaries such as insurance. Non-residents are charged at a much higher full cost recovery rate. Therefore this relatively simple payment administrative mechanism acts as a confirmatory check on the clinical utilisation statistics in IPAS. With this infrastructure in place, we believe that HA has virtually complete and accurate data capture of all inpatient episodes in the public sector and therefore can be taken as a reliable "gold-standard" in this audit exercise, much more so than in the case of most other countries with more complicated systems involving financial intermediaries, free care or otherwise unrecorded episodes.

The additional file 1 contains further technical details on survey sampling design, weighting methodology, questionnaire extracts and utilisation volume estimation formulae used.

### Statistical analysis

Territory-wide service utilisation volume as estimated from the 1999, 2001 and 2002 rounds of the THS was verified against corresponding statistics derived from the routine DH and HA administrative databases. The 2002 THS was undertaken between May and July, therefore the comparator period of July 2001 through June 2002 was adopted as the benchmark, to take into account possible seasonal effects of service utilisation. The comparator periods of observation were similarly determined for 1999 and 2001, albeit with six months as the duration of observation. (Table 2)

**Table 2:** Inpatient utilisation volumes derived from THS vs administrative data

	**1999**			**2001**			**2002**			**Cohen's d effect size index on under-reporting^§^**		
				
	**Admin data (a)**	**Survey data (b)**	**Net under-reporting 1- (b)/(a)**	**Admin data (a)**	**Survey data (b)**	**Net under-reporting 1- (b)/(a)**	**Admin data (a)**	**Survey data (b)**	**Net under-reporting 1- (b)/(a)**			
										**1999 vs 01**	**1999 vs 2002**	**2001 vs 2002**

**Comparator period**	May 1999 – Oct 1999	Sep – Nov 1999; last admission in past 6 months		Nov 2000 – Apr 2001	Jan – May 2001; last admission in past 6 months		Jul 2001 – Jun 2002	May – Jul 2002; all admissions in past 12 months				
**No. of persons ever discharged from public hospitals**	322,400 (295,974)*	205,039	36.4% (30.7%)*	345,029 (317,128)*	208,952	39.4% (34.1%)*	602,673 (546,793)*	337,868	43.9% (38.2%)*	1.64	3.88	2.20
**Total no. of discharges from private hospitals**	94,366	50,533^†^	46.4%	98,693	51,285^†^	48.0%	197,738	105,850	46.5%	0.49	0.007	0.59

* After excluding non-residents and deceased patients as at 31 Oct 1999, 30 Apr 2001 and 30 June 2002 respectively for the 3 rounds of THS

^†^Assuming one hospital episode only for those who reported a last admission to a private hospital in the past 6 months

§*Cohen's **for group 1 and 2 under comparison; where a = ad *min *istrative data, b = survey and *

We examined for differences in utilisation volumes between survey data and administrative statistics across corresponding years by calculating the Cohen's d effect size index, a standard statistical methodology, where a value of 0.2 indicates a small effect size, 0.5 a medium effect size and 0.8 or greater a large effect size [4].

To assess the potential for systematic biases in under-reporting, age- and sex-specific net under-reporting rates within each survey year were computed and the F-test was performed to evaluate differences between demographic subgroups at an overall significance level of 0.05. In addition, we modelled the effects of age and sex on the likelihood of ever hospitalisation in public hospitals by logistic regression to compare the odds ratios respectively derived from survey and administrative data. In the model based on survey data, individuals were dichotomised into ever and never hospitalised groups according to their survey responses. In the model using administrative data, the ever hospitalised group was defined based on HA's individual-level hospitalisation episode records whereas the never hospitalised group was derived from the difference between the territory-wide population figure and the former hospitalised headcounts. To evaluate the agreement between the two sources in the odds ratio of hospitalisation relative to a particular age subgroup as reference control, we assessed if there was overlap in the respective 95% confidence intervals of the odds ratios for each demographic subgroup. If there was no systematic difference in under-reporting between demographic subgroups, the odds ratios of hospitalisation for each age-sex subgroup derived from the survey data should be similar to the corresponding odds ratios derived from the administrative data. Lastly, an interaction term for age and sex was added to and tested for in the full models as health service utilisation might have varied in different gender and age groups. Although THS survey data contains other sociodemographic and patient characteristics which can be used for further comparison examining for systematic reporting bias by these variables, there is no corresponding information in the HA or DH routine databases that would allow for such.

All analyses were performed using SAS (SAS Institute Inc.) Version 8.0.

## Results

### Aggregate utilisation volumes in THS vs administrative data

Table 2 shows aggregate utilisation estimates derived from the THS in 1999, 2001 and 2002 and corresponding administrative databases for inpatient care episodes. The extent of net under-reporting was moderately large in all three years amounting to about one-third of all inpatient episodes, after adjustment by excluding deceased inpatients and non-residents during the survey periods from the denominator. Under-reporting appears to have been particularly acute in the 2002 round, perhaps due in part to the questionnaire design where only those who reported symptoms were asked about service utilisation, as opposed to documenting all care episodes regardless of the presence of symptoms. In addition, the recall period was longer (12 months vs six months) in the latest THS in 2002 as compared to the two previous rounds. Pairwise comparisons between years on the extent of net under-reporting, indicated significant differences between years, except for between 1999 and 2002 in terms of the total number of discharges from private hospitals. The magnitude of under-reporting was about one-third for inpatient episodes (30.7%, 34.1% and 38.2% respectively) after adjusting for non-Hong Kong residents (who were not covered by the THS) and those deceased as at the censor dates.

Net under-reporting was consistently higher for private compared to public sector inpatient admissions. This could have been an artefact where we had to assume only one hospitalisation episode for each person reporting a last admission in the previous six months whereas the DH administrative database contained information on the total number of discharges (i.e. not persons) from private hospitals. An individual with more than one hospitalisation episode would have generated only one count in the numerator but responsible for more than one in the denominator, thereby leading to artefactual under-reporting. In contrast, the HA database could accommodate person counts and therefore its data were directly comparable to the survey information. Moreover, due to the unavailability of detailed disaggregated data from the private hospitals, we could not adjust for deceased inpatients and non-residents in the estimation procedure.

### Differences in reporting by age and sex

As Table 3 illustrates, there does not appear to be significant systematic biases in the degree of under-reporting by age or sex. Within each survey year, we did not detect statistically significant differences between males and females except for inpatient episodes in 1999 (p = 0.04). Under the hypothesis of equal degree of under-reporting across all age subgroups, if the under five age group was excluded, there were no significant differences at the 0.05 level. On the other hand, if the under five age group was included, significant differences were found in 2002 (p = 0.02). This difference can likely be explained by proxy reporting for the under five age group.

**Table 3:** Extent of under-reporting (%) by age and sex in each THS

	**1999**	**2001**	**2002**
	
	No. of persons discharged from public hospitals in past 6 months*	No. of persons discharged from public hospitals in past 6 months*	No. of persons discharged from public hospitals in past 12 months*
***Age (years)***			
Less than 5	44.6%	51.8%	65.0%
5 – 24	36.2%	40.9%	38.2%
25 – 44	31.6%	31.9%	37.6%
45 – 64	27.3%	28.6%	38.0%
65 or above	25.7%	32.2%	31.7%
***Sex***			
Female	34.7%	36.3%	39.4%
Male	26.1%	31.4%	36.8%
**Overall**	**30.7%**	**34.1%**	**38.2%**
			
**F-test for the overall demographic subgroups differences within each year (at significance level 0.05)**^†^			
***Age difference***			
Including the 'less than 5' age group (p-value)	**0.47**	**0.18**	**0.02**
Excluding the 'less than 5' age group (p-value)	**0.53**	**0.28**	**0.60**
			
***Sex difference***(p-value)	**0.04**	**0.23**	**0.10**

* Adjusted for non-residents and deceased patients as at 31 Oct 1999, 30 Apr 2001 and 30 June 2002 respectively for the 3 rounds of THS

^† ^*where **a*_*i *_= *ad *min *istrative data*, *b*_*i *_= *survey data, for each demographic subgroup i up to k*.

**Figure 1:**
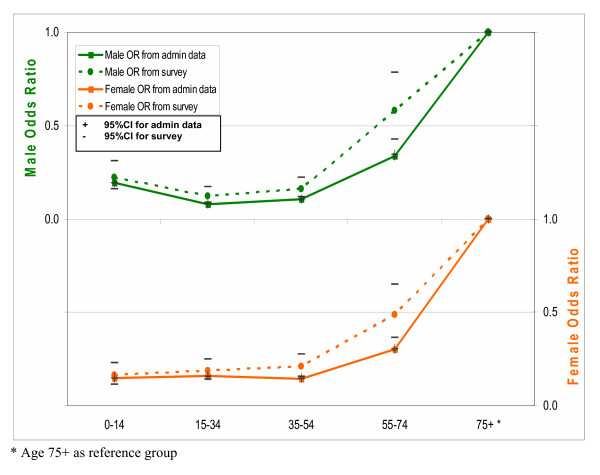
Comparison of odds ratios for age-sex effects on the likelihood of ever hospitalisation in public hospitals between administrative and survey data for year 1999.

**Figure 2:**
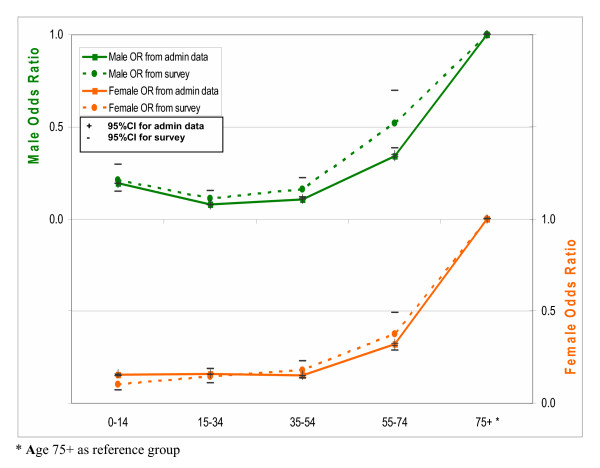
Comparison of odds ratios for age-sex effects on the likelihood of ever hospitalisation in public hospitals between administrative and survey data for year 2001.

**Figure 3:**
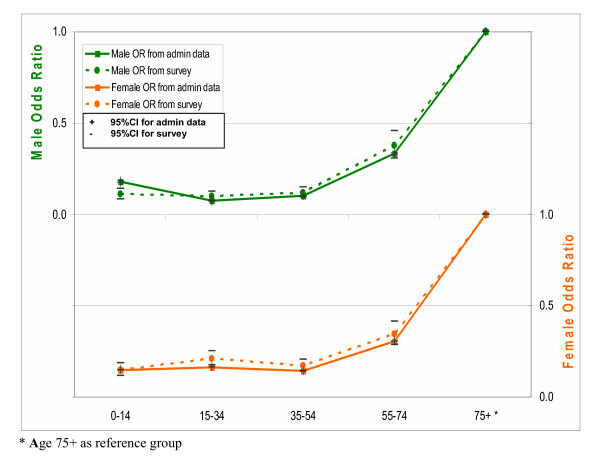
Comparison of odds ratios for age-sex effects on the likelihood of ever hospitalisation in public hospitals between administrative and survey data for year 2002.

As an alternative approach, we modelled the effects of age and sex on the likelihood of ever hospitalisation in public hospitals. The full model with the interaction term of age-sex was first fitted, but was subsequently dropped due to insignificant age-sex interaction effects. Figures 1, 2 and 3 plot age- and sex-specific odds ratios of ever hospitalisation and 95% confidence intervals (CIs) using both survey and administrative data. Both sets of curves are very similar in both direction and magnitude and largely overlap in their 95% CIs, confirming that the two data sources show consistent relativity in ever hospitalisation rate by age and sex. It suggests that there are no substantial systematic biases in under-reporting among age and sex subgroups.

## Discussion

In this large population-based audit, our findings show that under-reporting was consistently substantial, amounting to about one-third of all inpatient episodes, in the last three rounds of THS benchmarked against administrative data on the aggregate level. Differences in age and sex did not influence the degree of under-reporting.

Of note, although we observed under-reporting overall, the possibility that this represented mixed under- and over-reporting on the individual level cannot be ruled out. Under this ecologic design, it was not possible to disentangle the relative contributions of each type of recall error. The usual ideal study design would be to compare questionnaire-derived data with information abstracted directly from medical records on a person or episodic basis, thus allowing for more detailed analysis of the different types of reporting inaccuracy. Such a design would enable the quantification of recall error by measuring proportions of agreement and kappa values as well as the generation of 2 × 2 contingency tables and associated statistics such as true positives and negatives. However, this is prohibitively resource-intensive and cannot be routinely carried out for audit and benchmarking purposes, at least in the Hong Kong and other rapidly developing economy settings where such a management culture has yet to take hold. Moreover, widespread public concern about data privacy and the perception of possible government intrusion into personal medical and payment records in this laissez-faire society (in the politico-economic sense) would result in a low participation rate thus rendering the whole exercise useless. Therefore, the next best pragmatic alternative is to use aggregate statistics benchmarked against routine statistics as we have done here, especially when our unique circumstances and simple administrative and clinical care infrastructure particularly lend themselves to adopt such data as a reliable "gold-standard".

By this same reasoning, we did not consider outpatient episodes in this exercise. Provision of outpatient services is shared by both private and public sectors in the ratio of 70:30. While HA is responsible for all public specialist and general outpatient clinics and has the requisite data for comparison, the majority of ambulatory episodes are provided by private, self-employed, solo practitioners who charge on a fee-for-service basis and patients mostly pay out-of-pocket [5]. There is no central information repository for the private sector and only about one-third of such solo or small-group clinics are computerised which could potentially support an audit exercise [6]. Managed care, in the various forms of contract medicine, prepaid plans and preferred provider networks, has grown in the last decade although their penetration is still very limited in scope and size. About 30% of the population have private insurance or benefits schemes coverage, mostly through employment-based programmes. The majority of such coverage comes in the form of riders to other types of insurance schemes, most commonly life policies. Taking all these factors into consideration, an ecologic benchmarking exercise is not feasible for the outpatient sector.

Another potential caveat concerns the fact that private hospital data did not adjust for deceased and non-resident patients and multiple care episodes. However, HA hospitals accounted for 87% of all 34,237 deaths in Hong Kong in 2002 whereas the remainder took place in private hospitals (8.2%) and other places outside hospitals (4.8%). For the same year, non-residents only accounted for 0.5% of total inpatient episodes in HA hospitals. Therefore, it is unlikely multiple care episodes by the same individual can account for most of the under-reporting observed, especially given the very small market share of private hospitalisation.

A variety of reasons could potentially explain the under-reporting observed, including recall bias, non-response bias, sampling and estimation biases and questionnaire length and content. First, recall bias is the systematic over- or under-reporting of recall behaviour in surveys, such as health services use in this context. Respondents may forget relevant episodes or they may report an episode from outside the period of interest as if it had happened within the period (forward telescoping) or vice versa (backward telescoping). They may report episodes that do not meet the survey definition or they may fail to report relevant episodes because they perceive that such episodes do not meet survey criteria. For example, hospital transfers within the public sector are counted as two separate episodes in administrative databases but respondents usually only report them as a single episode. This has been addressed in the data analysis as both the administrative and survey data for the public sector were counted on a person basis. Deliberate backward telescoping would be possible if the respondent wishes to shorten the interview. Motivation to report tends to increase with saliency and frequency of events, and may decrease with increasing number of events. A final possibility regarding recall bias is that people genuinely forget. It seems amazing that anyone would forget hospital treatment. But other surveys as discussed in this paper have reached the same conclusion. Also there are similar findings in other areas of health. For instance, a study of recall of a diagnosis of cancer concluded that 20% of people forget that they have had cancer [7]. Recall bias is likely most responsible for the under-reporting observed in the THS.

Second, for the non-institutional sample in the 2002 round of THS, the overall non-response rate was 21.6%. A total of 2263 households could not be contacted after repeated visits and 503 households refused to respond. Non-response is usually more likely in high-income and singleton households. Unless the incidence and level of health services utilisation of non-respondents were substantially different from those who responded, this potential effect on the survey estimates would be limited especially in view of the current low rate of non-response. Assuming that the sample mean of the non-respondents were +10%, +30% and +50% of the corresponding mean of the respondents in the 2002 round of THS, the relative bias of non-response would have been -2%, -6% and -10% of the true population total of health services utilisation. Conversely, the relative bias would have been +2%, +7% and +12% respectively if the non-respondents' mean were -10%, -30% and -50% of the respondents' mean.

Third, sampling bias would be an issue if a non-representative survey sample results. This is highly unlikely in the THS series of surveys given the whole population coverage, explicit and validated sampling methodologies and the application of weighting factors to the results to ensure representativeness for the general population of Hong Kong (see additional file for details). Moreover, we have further improved the validity of the estimation procedure by excluding deceased inpatients and non-residents from the denominator in calculating the net under-reporting rate, thereby optimising the comparability between survey and administrative data.

Finally, unlike most other similar health statistics or utilisation surveys overseas, the THS series usually combines two or even three sub-surveys of disparate topics into an "omnibus" type of questionnaire for economy of scale and efficiency. Therefore the resulting survey instrument is often very long and can take up to 45 minutes to an hour to complete for each household. Coupled with the anecdotal observation that most Hong Kong residents maintain a very busy and hectic daily schedule, it is perhaps not surprising that respondents might have under-reported in order to complete the survey in a shorter period of time, although we know that in other settings overseas, interviews of 45 minutes to an hour are an acceptable burden to respondents. It might also have been quite difficult to focus on recalling specific details accurately when there are multiple topics covered in the same interview.

The extent of under-reporting as documented is moderate to large compared to experiences elsewhere. For instance, Harlow and Linet [8] in their systematic review found high proportions (at least 90% in three studies) of positive matches between records and survey interviews for hospitalisation episodes in four studies. However, some have criticised the design of those studies in which either positive survey responses were verified against medical records or positive record values were checked against survey responses, producing estimates which were biased towards either over- or under-reporting respectively. A full-design record check study, as recommended by Marquis [9], which identifies a population and sample from it independently of records, obtains survey and record information for each sampled element and compares the two data sources should be the "gold-standard", where both interview over-report (false positives) and under-report (false negatives) could be detected. The Health Interview Evaluation Survey (HIES) conducted by the US National Center for Health Statistics in 1990 [10], employed such a full design. It aimed to evaluate the accuracy of two-week doctor visit reporting through record checks. The study universe consisted of members of a staff model health maintenance organisation in Washington, D.C. The 1000 self-responding adult samples were selected from the membership roll, with an over-sampling of persons with recent ambulatory visits. Significant findings from the HIES, which were consistent with other findings in the literature, included: (i) under-reporting ranged about 17–35% and over-reporting about 20–40% for the 2-week reference period, but there was no evidence of general net under- or over-reporting of visits at the person level; (ii) under-reports were about 13–15% more prevalent for visits in the earlier week of the reference period than for those in the later week; (iii) under-reporting was greater for persons with more visits in the reference period; (iv) statistically significant differences in the percentage of positive match between household members present for the interview (84.4%) and those not present (46.9%) suggested some under-reporting by proxy respondents; and (v) males tended to under-report consistently more than females [10].

In comparison, Cartwright [11] also found under-reporting and over-reporting from adult self-respondents to be both about 21% in respect of physician contacts in a 4-week reference period "bounded" by interviews both at the start and at the end. The corresponding rates recorded in the study by Sudman et al [12], using a combined interview and diary procedure with a 3-month reference period, were 24% and 17% respectively. Means and Loftus [13] found rates of under-reporting and over-reporting in excess of 50% in respect of medical visits and hospital stays using a 1-year reference period.

Importantly, the HIES and other previous studies cited above indicate few consistent patterns of under- or over-reporting by respondents' demographic characteristics. Findings about age and health status were not consistent, and other characteristics were typically not associated with significant differences in reporting. This non-systematic recall pattern is also true in the present audit exercise. Age and sex (except for the under five age group in both sexes) did not appear to have influenced the extent of recall error on the aggregate level to any substantive degree. We did not have other data such as income and education level to examine for possible differential reporting behaviour, although there is little reason to believe these would be present given the lack of such effects seen in other studies and for age and sex in this study.

Therefore, the application of results such as relative measures of association between various socioeconomic and demographic characteristics and health care utilisation from such survey data for health planning (e.g. in formulating target subsidies for certain groups to achieve equity in health financing) in this context is reasonably valid, where random or non-systematic error would produce a conservative under-estimation of the true effect size. If the key interest is however in the absolute rate or volume for service planning use, the total service volume derived from survey estimates would need to be grossed up pro rata according to the degree of net under-reporting, as a crude measure to correct for such recall error.

## Conclusion

This audit has, for the first time in Hong Kong and elsewhere in Asia to the best of our knowledge, attempted to systematically ascertain the veracity and validity of health services utilisation estimates derived from household in-person interviews against routine administrative data. It is important that such an exercise be carried out on a regular basis as a continuous quality improvement initiative to ensure that data of the highest possible quality be used in the formulation of health care policy. Future research should explore the possibility of employing a full-design record check study to confirm the present findings and better understand other dimensions of recall and reporting behaviour. In addition, the current findings could be extended by analysing what sort of admissions people forget, e.g. are short stays less likely to be recalled compared to long stays? Are admissions for certain diagnoses not regarded as "proper" admissions and therefore not reported in surveys? Lastly, we should opt for psychological studies probing individuals for the mechanisms which suppress recall of an important health event.

## Competing interests

The author(s) declare that they have no competing interests.

## Authors' contributions

ELHT and GML conceived the study question and design. ELHT and PPSW carried out the statistical analysis, in consultation with GML. ELHT wrote the first draft and GML revised the manuscript. SC and SVL were responsible for the conduct and fieldwork of the Thematic Household Survey. All authors contributed to the critical evaluation of the methods, analysis and writing. GML is the guarantor of the study.

## Pre-publication history

The pre-publication history for this paper can be accessed here:



## Supplementary Material

Additional File 1 Technical details of Thematic Household Surveys in 1999, 2001 and 2002 – descriptions concerning the sampling design, weighting method, estimation formulae, questionnaire wording and survey respondent inclusion criteria are provided. Click here for file
