# Supplementary material for: Under-reporting of inpatient services utilisation in household surveys – a population-based study in Hong Kong
Source: BMC Health Serv Res. 2005 Apr 28;5:31. doi: 10.1186/1472-6963-5-31 (PMC1131900; doi:10.1186/1472-6963-5-31)
Supplement: Additional File 1 — Technical details of Thematic Household Surveys in 1999, 2001 and 2002 – descriptions concerning the sampling design, weighting method, estimation formulae, questionnaire wording and survey respondent inclusion criteria are provided. [file 1472-6963-5-31-S1.doc]

**Technical details of Thematic Household Surveys in 1999, 2001 and 2002**

1. **Sampling design**

The 2002 round of THS covered the entire land-based population of Hong Kong including both institutional and non-institutional residents but excluded those hotel transients and persons living on board vessels. For the 1999 and 2001 rounds, only non-institutional residents were covered.

For all three rounds of survey alike, the sample of non-institutional residents was selected by the C&SD from the Register of Quarters (RQ) in Hong Kong. The Register consists of a list of addresses of permanent quarters in built-up areas throughout all districts in Hong Kong. Each unit of quarter is identified by a unique address with details on the house number, street name, building name, floor number and flat number. Temporary structures like roof-top and backlane structures are usually not listed in RQ, but each is treated as an attachment to the nearest listed quarter in the RQ. Records of quarters in the RQ were first sorted by geographical area and type of quarters. Systematic replicated sampling was then applied in which sampling units were drawn with a fixed interval after selecting a random start to form sample replicates. The size of each sample replicate was about 490 quarters. All households within the sampled quarters and all persons within the households, except the domestic maids, were approached for face-to-face interview. The number of households and persons successfully interviewed and the response rate for the three surveys are given in Table 1 of the paper.

Regarding the institutional residents sample in the 2002 round, a stratified two-stage cluster sampling was adopted. At the first stage, a total of 871 institutions providing residential services were stratified into 6 types of services (government subvented and private elderly homes; rehabilitation and medical social services for blind, physical handicap, mentally handicapped and mentally ill) and further into two enrolment sizes (<=100 and 100+). Within each of the 11 strata, the institutions were systematically selected based on a target sampling fraction of 10%, or at least two institutions, whichever was larger. At the second stage, a target sampling fraction of 3.3% was applied to select residents from each sampled institution, or at least 30 residents per institution, whichever was larger. The residents were selected systematically with a random start on the basis of the name list or bed numbering system of the sampled institutions. Out of the total 92 institutions being sampled, 2,111 persons were successfully interviewed.

1. **Weighting method**

The survey data collected for non-institutional residents were weighted in accordance with the number of persons by sex, age and housing type in the land-based non-institutional population to derive the overall population estimates. The entire land-based non-institutional population N was stratified into strata Nh′s in accordance with gender, 14 age groups and two housing types, and each weight was calculated by dividing the total number of persons in stratum h (Nh) by the number of persons interviewed in that stratum (nh). There were 56 weighting factors defined for the non-institutional respondents. This weighting method was consistently applied in all rounds of THS.

According to the stratification in sample selection for the institutional population, each interviewed person of institutional population was weighted up by the ratio of the population figure in stratum h to the number of interviewed persons in that stratum (N_h_/n_h_). There were 11 weighting factors defined for the institutional respondents.

1. **Estimation formula**

The utilization volume of a particular type of services in the recall period was estimated as follows. It includes number of episodes in public hospitals and number of episodes in private hospitals.

*For each weighting stratum h:*

where

= Estimated total utilization volume e.g. no. of inpatient episodes

$N_{h}$ = Population

$n_{h}$ = No. of sampled persons (successfully interviewed)

$n_{ha}$ = No. of sampled persons who report to have used the particular services within a specified period

$\sum_{n_{ha}} y_{h}$ = Sum of total utilization volume within a recall period over those sampled persons who have ever used the service

1. **Questionnaire wording**

Survey data were collected through the use of structured questionnaires. In order to test the applicability of the questionnaires and the fieldwork procedures, a pilot survey was conducted before the formal fieldwork.

In the 2002 round of THS, the question regarding hospitalization was asked among all survey respondents:

Have you stayed in hospital in Hong Kong in the past 12 months? Please only include those cases which you had to make registration and disregard whether you had to stay overnight. In the past 12 months, how many times have you been admitted to hospital under Hospital Authority? And private hospital? Which HA hospital(s) and private hospital(s) have you stayed? (Probe the name and department)

The wording was identical in the 1999 and 2001 rounds, except that the recall period was changed to six months and only details of the last hospital admission were asked.

1. **Survey respondents**

For persons aged 12 and below and those who were mentally unfit to respond to the survey, their primary care-takers were requested to provide proxy answers for them except for the self-report health status questions. For the institutional residents, the survey responses were often collected through proxy reporting instead of self-reporting. For the non-institutional residents, proxy reporting was allowed for member(s) in a household who were not present at repeated household visits by interviewers.
